# Zoonotic sources and the spread of antimicrobial resistance from the perspective of low and middle-income countries

**DOI:** 10.1186/s40249-023-01113-z

**Published:** 2023-06-14

**Authors:** Ioana D. Olaru, Birgit Walther, Frieder Schaumburg

**Affiliations:** 1 Institute of Medical Microbiology, University of Münster, Münster, Germany; 2 Advanced Light and Electron Microscopy, Robert Koch-Institute, Berlin, Germany; 3 Department of Environmental Hygiene, German Environment Agency, Berlin, Germany

**Keywords:** Antimicrobial resistance, Extended-spectrum Beta-lactamase, Methicillin-resistant *Staphylococcusaureus*

## Abstract

**Background:**

Antimicrobial resistance is an increasing challenge in low and middle-income countries as it is widespread in these countries and is linked to an increased mortality. Apart from human and environmental factors, animal-related drivers of antimicrobial resistance in low- and middle-income countries have special features that differ from high-income countries. The aim of this narrative review is to address the zoonotic sources and the spread of antimicrobial resistance from the perspective of low- and middle-income countries.

**Main body:**

Contamination with extended-spectrum beta-lactamase (ESBL)-producing *Escherichia* *coli* is highest in poultry (Africa: 8.9–60%, Asia: 53–93%) and there is a risk to import ESBL-producing *E.* *coli* through poultry meat in Africa. In aquacultures, the proportion of ESBL-producers among *E.* *coli* can be high (27%) but the overall low quality of published studies limit the general conclusion on the impact of aquacultures on human health. ESBL-producing *E.* *coli* colonization of wildlife is 1–9% in bats or 2.5–63% birds. Since most of them are migratory animals, they can disperse antimicrobial resistant bacteria over large distances. So-called ‘filth flies’ are a relevant vector not only of enteric pathogens but also of antimicrobial resistant bacteria in settings where sanitary systems are poor. In Africa, up to 72.5% of ‘filth flies’ are colonized with ESBL-producing *E.* *coli*, mostly conferred by CTX-M (24.4–100%). While methicillin-resistant *Staphylococcus aureus* plays a minor role in livestock in Africa, it is frequently found in South America in poultry (27%) or pork (37.5–56.5%) but less common in Asia (poultry: 3%, pork: 1–16%).

**Conclusions:**

Interventions to contain the spread of AMR should be tailored to the needs of low- and middle-income countries. These comprise capacity building of diagnostic facilities, surveillance, infection prevention and control in small-scale farming.

**Graphical abstract:**

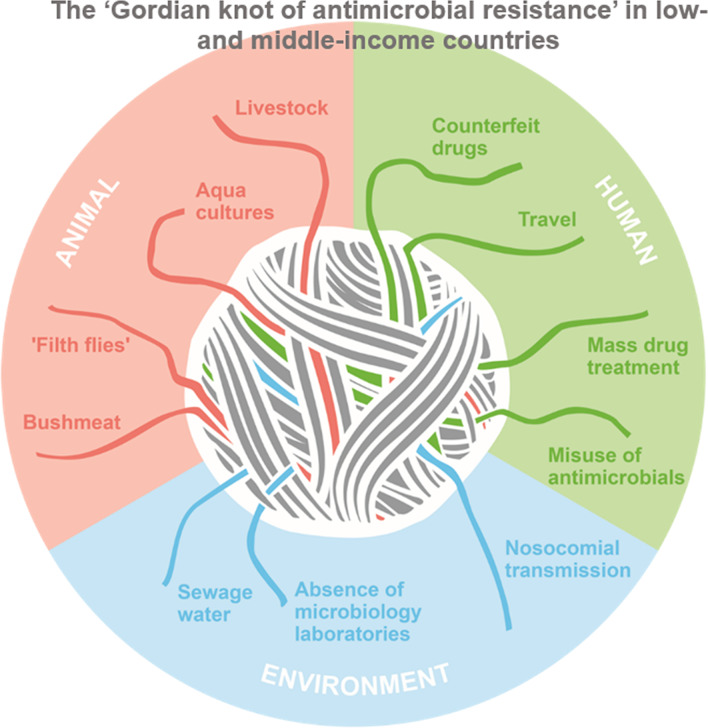

## Background

The increase in antimicrobial resistance (AMR) poses a considerable threat to health and livelihoods. An estimated 4.95 million deaths associated with AMR occurred in 2019 [1]. Most AMR associated deaths occur in low- and middle-income countries (LMIC) and are therefore linked to poverty, income inequalities, difficulties in healthcare access, and inadequate or lacking policies for preventing the development and transmission of AMR [1–3]. AMR is, however, a global problem affecting both high-income and LMIC alike. Beyond the direct effect on human health, AMR in livestock can affect livelihoods particularly among the most vulnerable people [4].

The aim of this narrative review is (i) to shed light on the various zoonotic sources from which resistant bacteria can be transmitted in LMIC, and (ii) to address multilateral health policies to contain this spread effectively. This work addresses AMR in the context of food production from animal rearing (livestock and aquaculture) as well as at the interface between humans and wildlife. Specifically we are discussing the prevalence of resistant bacteria that may lead to transmission and dissemination either directly from animal contact or consumption of animal products (e.g. AMR in wildlife and in wild animals used for food), or indirectly (e.g. through ‘filth flies’). We mainly focus exemplarily on extended-spectrum beta-lactamase (ESBL)-producing *Escherichia* *coli* and methicillin-resistant *Staphylococcus* *aureus* (MRSA) in LMIC (excluding China) and the most important pathogens associated with aquaculture as these are of critical importance for understanding AMR particularly in the community setting in LMIC. China was largely excluded as this topic has recently been reviewed elsewhere [5]. For country income classification, the definitions from the World Bank were used [6].

### Antimicrobial resistance: mechanisms and transmission

Antimicrobial agents either inhibit (bacteriostatic agents, e.g., macrolides, lincosamides) or kill bacteria (bactericidal agents, such as beta-lactams). Beta-lactam antimicrobials (e.g. penicillins, aminopenicillins, cephalosporins) inhibit the synthesis of the peptidoglycan layer of the cell wall. Resistance to beta-lactams is mediated either by the expression of bacterial enzymes that lyse the beta-lactam ring in Gram-negative rods (beta-lactamases) or by alterations of the cellular target, e.g. penicillin-binding proteins in Gram-positive cocci. In Enterobacterales (e.g., *Escherichia* *coli*), beta-lactamases, such as ESBL and/or AmpC beta-lactamases can hydrolyse not only narrow-spectrum penicillins, but also cephalosporins and beta-lactam/beta-lactamase inhibitor combinations. AMR can result through mutations of chromosomal or extrachromosomal genes or the acquisition of resistance genes from other organisms. Selective pressure such as that exerted by antimicrobial treatment, leads to the generation, survival, and proliferation of resistant clones and can promote the exchange of resistance determinants within and between bacterial species [7]. Plasmids are of particular importance as they can carry resistance genes that confer resistance to multiple antimicrobial classes and are responsible for the global dissemination of resistance to key antimicrobials such as carbapenems and third-generation cephalosporins [8–12]. Genetic material can also be exchanged by transformation or transduction via bacteriophages [7].

### Drivers of antimicrobial resistance

**Fig. 1:**
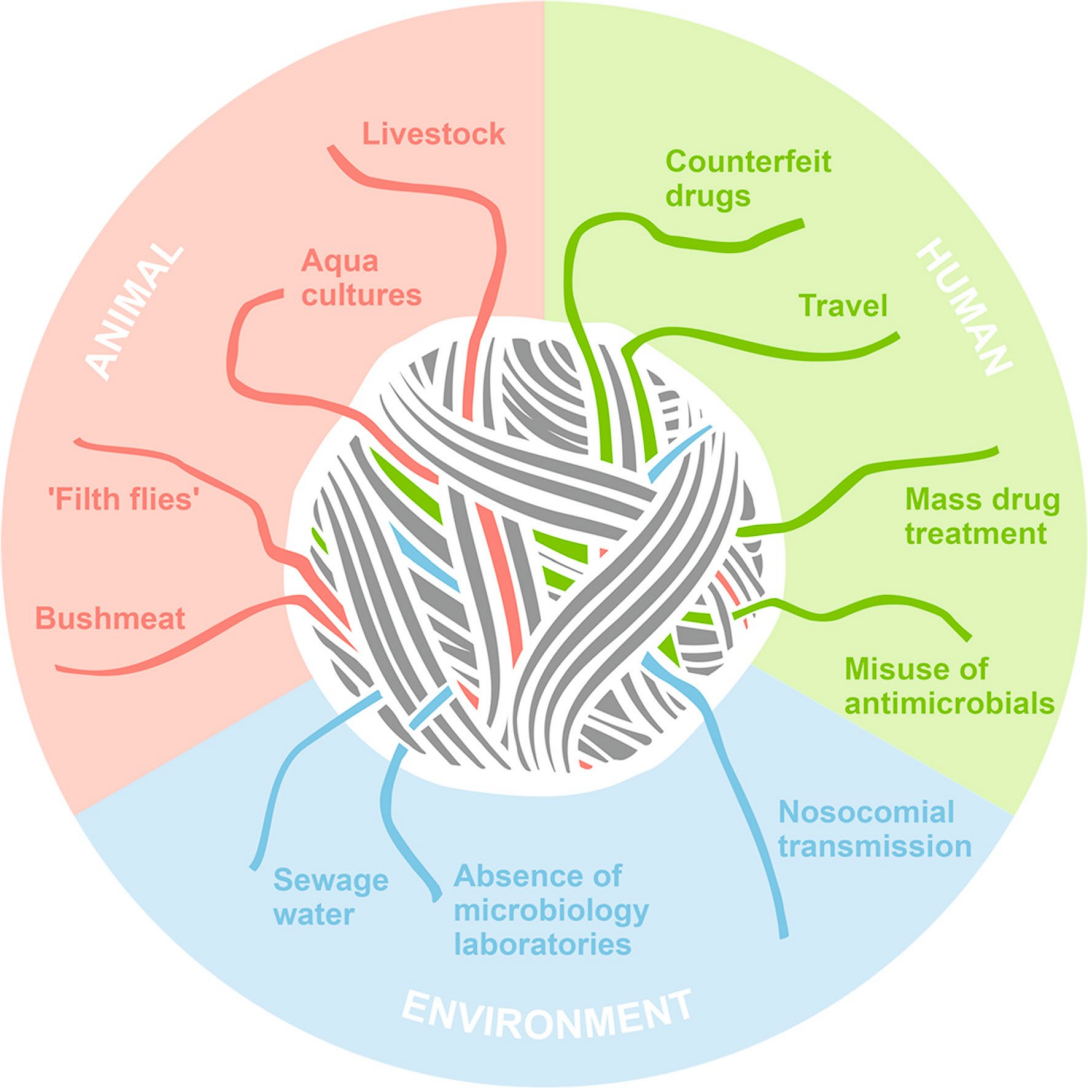
The ‘Gordian Knot of antimicrobial resistance’ in low- and middle-income countries (LMIC) describing factors that contribute to the transmission of antimicrobial resistance at the human-animal interface. Reservoirs and sources of antimicrobial resistance (AMR) are in the environment, in animals and humans, which is considered in the One Health concept. This review focuses on zoonotic reservoirs and sources in LMIC. Additional drivers of AMR in LMIC are related to human health (travel [157], mass drug treatment, e.g. for trachoma [158], misuse of antimicrobials [7], counterfeit drugs [159], hospital transmission, absence of microbiological laboratories) and the environment (sewage water [160])

The development, selection, and transmission of AMR can be attributed to multiple factors. Inappropriate and excessive antimicrobial use (AMU) are well-recognised drivers of AMR (Fig. 1) [7]. In humans, antimicrobial consumption has increased by 65% between 2000 and 2015 [13]. This increase has been driven in particular by LMIC and is closely related to increases in gross domestic product [13]. Beyond their use for treating infections in animals, antimicrobials are used for prophylaxis and growth promotion sometimes as measures for counteracting lacking hygiene [4]. Antimicrobials used in animals often belong to the same classes as those used in humans and transmission of resistant bacteria between animals and from animals to humans has been extensively reported.

According to the World Organization for Animal Health (WOAH) report, 69,455 tonnes of antimicrobial agents were used in animals in 2018 [14]. The highest antimicrobial quantities, adjusted for animal biomass, were used in countries from Asia and the lowest in Africa. Encouragingly, a decrease by 27% was observed in antimicrobials used in animals between 2016 and 2018 although most countries did not report AMU data for estimating time trends [14]. Similarly, fewer countries than in previous years reported on using antimicrobials for growth promotion [14]. This is in line with the WHO recommendations to completely restrict the use of antimicrobial drug classes important to human health for growth promotion in animals [15]. It is hoped that AMU in food production will decline in the next years due to the implementation and enforcement of regulations restricting use, a decrease in meat consumption, and user fees for AMU in food-production animals [4]. However, the reliability of AMU estimates can be uncertain. Other authors estimate that AMU in animals will in fact increase by 8% between 2020 and 2030, when it will reach 107,472 tonnes with Asia accounting for 67% of the global use and Africa for less than 1% [16]. A recent scoping review highlighted the lack of standardization of data from research studies on AMU in animals [17]. Furthermore, participants were occasionally unable to differentiate antimicrobials from other substances suggesting a lack of reliability of reported data [17]. Interventions that restrict the use of antimicrobials in animals have been shown to reduce the burden of AMR in animals and are likely to also reduce AMR in humans [18].

Antimicrobials and their residues can accumulate in the environment through contamination with human or animal waste, inadequate disposal of waste products resulting from the manufacture of antimicrobials, and the use of antimicrobials as pesticides (Fig. 1) [19]. Contact of animals with contaminated water and soil leads to transmission of resistant bacteria to and from food animals and within food chains. In addition, other factors such as lacking access to diagnostics, healthcare transmission, mobility of humans (e.g. travel), goods, freight, foods and plants further contribute to the transmission of resistant organisms [7]. A well-known example of global dissemination resulting from travel is that of the plasmid-mediated colistin resistance conferred by the gene *mcr*-*1* [20]. Plasmid-transmitted *mcr-1* was first described in 2016 in Southern China, and likely resulted from the use of colistin for growth promotion in animals [10]. In the following years, the presence of *mcr-1* has been reported in multiple bacterial species across the world [12]. Furthermore, imported meat [21, 22] and wildlife [23] have also been reported to carry AMR across borders.

## Main text

### Livestock for food production

A source for transmission to humans can be antimicrobial resistant bacteria in livestock such as livestock-associated MRSA (LA-MRSA), or ESBL-producing Enterobacterales [24, 25]. The extent and direction of transmission is, however, often unclear as longitudinal genotyping studies are largely missing to track transmission events, particularly in LMIC [26]. Therefore, transmission is often assumed if resistant isolates from animals and humans share indistinguishable genomic profiles without knowing the direction of transmission. While livestock in LMIC can be colonized with antimicrobial resistant pathogens, transmission most likely occurs while handling livestock products such as meat. In the following section, we therefore focus on the contamination of meat and other animal products for consumption and largely do not consider faecal colonisation studies of livestock.

### Africa

In Africa, the contamination of meat with ESBL-producing *E.* *coli* depends on the animal species and is highest in poultry (8.9–60%) [27–29] followed by pork and beef (2.3–22%, Table 1) [30–32]. In sub-Saharan Africa, poultry meat is often imported from high-income countries (e.g. USA, Europe) and particularly poultry from Europe and Brazil (Table 1) is contaminated with ESBL-producing *E.* *coli* (up to 54%) [29, 33–35]. Whether imported poultry meat can be a source for colonisation with ESBL-producing bacteria in humans is controversial as cefoxitinase CTX-M subtypes in poultry meat (*bla*_CTX-M-1_, *bla*_CTX-M-14_) differed from those in humans (*bla*_CTX-M-15_) in one study from Gabon [29]. In contrast, a study on faecal samples in Ghana revealed that locally raised poultry and hospitalized patients share the same lineages of ESBL-producing *E.* *coli* (mainly *bla*_CTX-M-15_, sequence type [ST]38 and ST58) suggesting transmission or the spread of global clones both in animals and humans [36]. Another study from Ghana showed that imported poultry is less frequently contaminated with ESBL-producers than locally produced poultry (31 vs. 44%) [33]. Meat from LMIC can also be exported to high income countries: Up to 95% of broiler meat from Brazil was contaminated with ESBL/AmpC**-**producing *E.* *coli* on the Swedish market (AmpC is a beta-lactamases that hydrolyse penicillins, and second- and third-generation cephalosporines) [21]. Similarly, 29.5% of chicken meat batches imported to the UK from South America were contaminated with ESBL-producers mostly deriving from Brazil (*bla*_CTX-M-2_, *bla*_CTX-M-8_) [37]. Thus, international poultry trade can pose a risk to import or export ESBL producing Enterobacterales. There is evidence that the “pathogen reduction treatment” with chlorinated water in the US can reduce the risk of contamination with ESBL-producers in poultry [29, 38]. Here, eviscerated carcasses are washed with chlorinated water to remove harmful bacteria such as *Salmonella* sp.

**Table 1:** Contamination of livestock meat with ESBL-producing *Escherichia* *coli*

Region	Major resistance genes	Year	Country	Samples (*n*)	Prevalence [% (*n*/*N*)]	References
Africa	*bla*_CTX-M-14_, *bla*_CTX-M-15_	2013	Egypt	Poultry (112)	8.9% (10/112)	[27]
	Not done	2016	Ethiopia	Beef (88)	6% (5/88)	[126]
	Not done	2020	Ethiopia	Beef (556)	2.3% (13/556)	[30]
	*bla*_CTX-M-1_, *bla*_CTX-M-14_	2011–2012	Gabon	Poultry (60)	23% (14/60)	[29]
	*bla*_CTX-M-15_	2013	Ghana	Poultry (188)	10.6% (20/188)	[34]
	*bla*_CTX-M-1_, *bla*_CTX-M-14_, *bla*_CTX-M-15_	2015	Ghana	Poultry (200)	23% (46/200)	[33]
	*bla*_TEM_	2019	Ghana	Goat (108), beef (81), sheep (16)	2% (4/205)	[127]
	*bla*_CTX-M-164_	2015	Mozambique	Poultry (99)	17% (17/99)	[35]
	Not applicable	2009–2014	Nigeria	Poultry (unknown)	0% (0/unknown)	[128]
	*bla*_CTX-M-1_, *bla*_CTX-M-8_, *bla*_CTX-M-14_, *bla*_TEM-1b_, *bla*_SHV-5_	2006	Tunisia	Beef (23), poultry (10), sheep (1), fish (4)	29% (11/38)	[32]
	Not applicable	2004–2005	Tunisia	Sheep (8), poultry (7), beef (4), fish (3), pork (1)	0% (0/23)^a^	[129]
	*bla*_CTX-M-1_, *bla*_TEM-1b_, *bla*_TEM-20_	2007	Tunisia	Sheep (28), poultry (26), beef (14), fish (10), horse (1)	13% (10/79)	[31]
	Not applicable	Before 2009	Tunisia	Poultry (55)	0% (0/55)^a^	[130]
	*bla*_CTX-M_	Before 2016	Zambia	Poultry (384)	20.1% (77/384)	[28]
South-East Asia	*bla*_CTX-M_	2016	Cambodia	Pork (60)	75% (45/60)	[43]
	*bla*_CTX-M_	2016	Cambodia	Poultry (30)	53% (16/30)	[43]
	*bla*_CTX-M-15_	2014–2015	Cambodia	Pork (110)	0% (0/110)	[45]
	*bla*_CTX-M-15_	2014–2015	Cambodia	Poultry (87)	0% (0/87)	[45]
	*bla*_CTX-M-15_	2014–2015	Thailand	Pork (175)	4% (7/175)	[45]
	*bla*_CTX-M-15_	2014–2015	Thailand	Poultry (189)	0% (0/189)	[45]
	*bla*_TEM_, *bla*_SHV_, *bla*_CTX-M_	2012–2013	Malaysia	Poultry (160)	54% (86/160)	[131]
	*bla*_CTX-M_, *bla*_TEM_, *bla*_SHV_	Before 2022	Pakistan	Poultry and livestock (250)	30% (75/250)	[132]
	*bla*_*TEM*_	Before 2022	Thailand	Minced meat (150)	52% (78/150)	[133]
	*bla*_CTX-M_, *bla*_TEM_, *bla*_SHV_	2012–2014	Vietnam	Poultry (82)	93% (76/82)	[44]
	*bla*_CTX-M_, *bla*_TEM_, *bla*_SHV_	2012–2014	Vietnam	Pork (92)	35% (32/92)	[44]
	*bla*_CTX-M_, *bla*_TEM_, *bla*_SHV_	2012–2014	Vietnam	Beef (74)	34% (18/74)	[44]
	*bla*_CTX-M-1_, *bla*_CTX-M-9_, *bla*_TEM_,	2015–2017	Vietnam	Poultry (116)	66% 77/116	[47]
	*bla*_CTX-M-1_, *bla*_CTX-M-9_, *bla*_TEM_,	2015–2017	Vietnam	Pork (112)	55% (62/112)	[47]
	*bla*_CTX-M_, *bla*_TEM_, *bla*_SHV_	Before 2022	Vietnam	Poultry (60)	90% (54/60)	[46]
Latin America	*bla*_CTX-M-2,_ *bla*_CTX-M-15_	2014	Brazil	Poultry (100)	6% (6/100)	[56]
	*bla*_CTX-M-2,_ *bla*_CTX-M-55_	2019	Brazil	Poultry (50)	42% (21/50)^a^	[58]
	*bla*_CTX-M-2,_ *bla*_CTX-M-55_	2019	Brazil	Pork (50)	12% (6/50)	[58]
	*bla*_CTX-M-2,_ *bla*_CTX-M-8_*,* *bla*_CTX-M-14_*,* *bla*_CTX-M-55_	2018–2019	Brazil	Lamb (25)	56% (14/25)	[134]
	*bla*_CTX-M-3,_ *bla*_CTX-M-55_	2017–2018	Ecuador	Poultry (335)	72.8% (244/335)^a^	[57]

This table focuses on pathogens isolated from meat. It is anticipated that faecal samples from livestock would yield similar levels of colonization with ESBL-*E.* *coli*

^a^Deduced from third generation cephalosporin resistance

MRSA is less commonly detected in meat products in Africa and contamination rates are estimated to be 7.8% in a recent review with highest contamination rates in pork (12%) followed by poultry (6.8%) and beef (6.1%) [39]. While LA-MRSA clonal complex (CC) 398 is common in Europe, particularly in pork (up to 25%) [40, 41], it is rarely detected in Africa. So far, LA-MRSA CC398 in meat was only reported in Tunisia (poultry, veal) [42].

### Asia

In Asia, the prevalence of ESBL-producing *E.* *coli* in meat samples and livestock is considerably higher than in Africa (Table 1). ESBL-producing *E.* *coli* was isolated from 53–93% chicken meat samples [43, 44] and 35–75% pork meat [43, 44]. In contrast, one study conducted in Thailand and Cambodia reported very low prevalence of contamination of less than 4% for both poultry and pork [45]. In addition to ESBL, meat products from Asia are relatively often contaminated with organisms harbouring *mcr-1* genes [46, 47].

Conversely, the prevalence of MRSA is relatively low in livestock and animal products in Asia. MRSA was found in 3% of chicken meat samples, but not in pork or goat meat in India [48]. In contrast, in a study from Pakistan, MRSA was isolated from 11% of eggs from retail shops and all isolates were positive for the Panton-Valentine leukocidin (PVL) [49]. PVL is produced by some *S.* *aureus* strains and is a virulence factor leading to cell lysis and tissue damage. PVL is widespread in the tropics, particularly in Africa and associated with severe skin and soft tissue infections [50].

While CC398 is a common MRSA clonal complex in livestock in Europe, CC9 is the predominant MRSA clone in pig farming in Asia [51, 52]. In Sri Lanka, MRSA prevalence in pigs was considerably higher than in poultry or cattle (16% vs. 9.3% and 6.2%, respectively) [53]. A higher prevalence of MRSA among pig farmers than among poultry and cattle farmers was also observed suggesting possible transmission of MRSA between humans and pigs [53]. Several studies from Thailand also report on the prevalence of MRSA colonization among pigs and pig farmers. A study investigated 104 pig farms from two provinces in Thailand and found that in almost 10%, MRSA could be isolated from pigs, farmers, or the farm environment [54]. Among individual pigs, 2.5% were MRSA-colonised, all with ST9 clones, harbouring staphylococcal cassette chromosome (SCC)-mec IV [54]*.* In another study from the same country, the prevalence of LA-MRSA was less than 1% [52].

### Latin America

Latin America, particularly Brazil and Argentina, is a major meat exporting region providing beef and poultry for the African and Asian markets. While numerous studies analysed the burden of AMR in humans, comparably little is known on AMR in food items such as meat in South America [55]. Contamination rates of poultry meat with ESBL-producing *E.* *coli* (6–72.8%) are comparable with other LMIC (Table 1) [56–58]. Not only ESBL but also plasmid-mediated AmpC beta-lactamases can be widespread in *E.* *coli* from Brazilian poultry meat [59]. It is therefore not surprising, that ESBL/AmpC-producing *E.* *coli* are disseminated through meat from South America to Europe and to LMIC as outlined above.

Poultry meat from South America has the highest MRSA prevalence in the world (27% vs. 1% in North America) [60]. This is in line with contamination rates reported for pork meat, which are also much higher in Brazil (37.5%) [61] and Chile (43.1–56.5%) [62] compared to Africa and South-East Asia. In conclusion, the global meat trade can promote the spread of AMR (e.g. ESBL-*E* *coli*, MRSA) from, in and between LMIC.

### Aquaculture for food production

According to the Food and Agriculture Organization (FAO), aquaculture is defined as the farming of aquatic organisms including fish, molluscs, crustaceans and aquatic plants in inland and coastal areas [63]. In 2020, the global aquaculture production was estimated at 87,501 thousand tonnes of live animal weight with the Asian region accounting for 88% of the total production (77,377 thousand tonnes) and including a considerably high production in China contributing 56.7% [63]. Production from the Latin American and African regions amounted to 3781 (4%) and 2354 (3%) thousand tonnes, respectively [63]. It has been discussed whether aquaculture has the ability to improve livelihoods and health in LMIC by contributing to the progress of a number of inter-related sustainable development goals (SDG) such as SDG2 “zero hunger” and SDG3 “good health and well-being” [64, 65]. In 2020, aquaculture production reached an all-time record of 214 million tonnes including 178 million tonnes of aquatic animals [63]. The rapid growth in aquaculture production in recent decades has been facilitated by a transition from extensive to intensive farming [63, 66, 67]. Hence, multiple changes have arisen from increased aquaculture production that include, among others, major adverse effects associated with improper site selection, use of chemicals and anti-infective agents, increased land use, and a global increase of inland water use from 12% (in the late 1980s) to 37% in 2020 [63, 64, 66–68]. Since intensified aquaculture is inevitably associated with disease occurrence in large-scale animal production settings, antimicrobials are used for therapeutic and prophylactic purposes [69–71]. In many countries, aquaculture production systems are not separated from the environment leading to the accumulation of antimicrobial residues in the waters used for animal farming and adjacent waters affecting wild fish, plankton and sediments [71, 72]. This leads to the selection of antimicrobial resistant bacteria and changes the composition of environmental bacteria [67]. The number of antimicrobial substances approved for the use in aquaculture varies markedly between different countries (e.g. two in Brazil, 30 in Vietnam) [73]. The technical guidelines on the prudent and responsible use of veterinary medicines in aquaculture (provided by the FAO) address the need for appropriate environmental assessment and monitoring of drug and chemical use and its impact [74]. However, standardized AMU indicators for aquaculture are not available, counteracting any efforts to compare antimicrobial use on a local or global scale [75].

A recent systematic review and meta-analysis of 749 point-prevalence surveys published between 2019 and 2000 and reporting on AMR bacteria from aquatic food animals intended for human consumption in Asia revealed concerning levels of resistance to medically important antimicrobials in foodborne pathogens such as *Vibrio* sp*.*, *Aeromonas* sp., *Streptococcus* sp., *Edwardsiella* sp. and *E.* *coli* [66]. The overall prevalence of resistance to the highest priority critically important antimicrobials for human medicine defined by WHO [76] were 34% for macrolides, 18% for third- and fourth-generation cephalosporins and 16% for quinolones [66]. For *E.* *coli*, the prevalence of resistance to third-generation cephalosporins (indicative ESBL/AmpC production) was 27%, 10% to fosfomycin and 5.2% to colistin across the investigated Asian sub-regions [66]. Other studies from Southeast Asia reported that ESBL-producing *E.* *coli* was isolated from 20–53% of fish and shrimp samples [43, 44, 47].

However, available studies on AMR among the most important pathogens associated with aquaculture or its products have considerable differences regarding study design, strategy for analysis, sampling procedures, methods for pathogen identification (species level), and characterisation that often hinder a systematic comparative analysis [77]. The frequent occurrence of significant errors regarding testing methodologies, quality controls, and the use of appropriate interpretive criteria in the performance and reporting of susceptibility testing results of bacteria isolated from aquatic animals were recently addressed [78]. Chromosomal genes conferring resistance towards beta-lactams in *Aeromonas* spp. [79] led, for instance, to the recommendation to consider respective isolates from human clinical samples (i.e. members of *Aeromonas* *caviae* complex, *Aeromonas* *hydrophila* complex, and *Aeromonas* *veronii* complex) as uniformly resistant to ampicillin, amoxicillin-clavulanic acid, and cefazolin [80]. It is important to consider expected resistant phenotypes, formerly ‘intrinsic resistances’, when reporting prevalence, especially when combining results for multiple species, because results can be misleading and, may therefore interfere with our general understanding regarding the origin and spread of AMR [81]. The methods used for antimicrobial susceptibility testing of *Vibrio* sp. associated with aquaculture were recently analysed and revealed, that although 203 studies reported on the prevalence of resistance, 185 of them did not provided the criteria they used to determine resistance, used criteria that had not been validated, or were inappropriate [77].

A recent review highlighted that, although most WHO member states have developed a national action plan on AMR compliant with the “One Health” perspective, almost 40% do not acknowledge aquaculture as a critical component where AMR should be further investigated and contained along the whole production chain [82]. Hence, aquaculture will continue to pose challenges in terms of the rapid dissemination of antimicrobial resistant pathogens and AMR determinants since the aquatic environment provides a conducive environment for drug residues, microbial pathogens, and antimicrobial resistance gene dispersions [71].

### AMR in wildlife and bushmeat

Bushmeat or wild meat from non-domesticated animals (e.g., bats, monkeys, reptiles, squirrels) is, apart from livestock meat and aquaculture, an important protein source in the Global South. Approximately 5 million tonnes of bushmeat [83] are consumed annually which is the same amount of meat produced in Canada in 2020 (5.2 million tonnes) [84]. The handling and consumption of bushmeat is a risk factor for the transmission of (emerging) zoonotic diseases (e.g. Ebola, *Rickettsia*, *Brucella*, mpox) and can expose humans to antimicrobial resistant bacteria.

AMR in wildlife is mostly an indicator of environmental contamination (e.g., surface water, food) with antimicrobial resistant bacteria [85]. For instance, colonization with antimicrobial resistant bacteria in rats and shrews was significantly higher in the vicinity of farms than in more remote areas in Vietnam (forest or rice paddies) [86].

Latest models suggest that the risk for exposure to emerging infectious diseases caused by drug resistant pathogens in LMIC is highest in West Africa and East Asia [87]. Similar to livestock meat, *E.* *coli* and *S.* *aureus* are the most relevant antimicrobial resistant Gram-negative and Gram-positive species in bushmeat [88]. Apart from one study in Peru [89], transmission of ESBL-producing *E.* *coli* between wild animals/bushmeat and humans or livestock have not yet been traced, but numerous observations suggest a certain role of bushmeat/wildlife in the spread of AMR. For instance, bats (1–9%) or birds (2.5–63%) are among the major species to be colonized with ESBL-producing organisms (Table 2), they live in proximity to humans (e.g. *Eidolon* *helvum,* *Fregata* *magnificens*) [90–92] and are very mobile and could therefore spread pathogens over large distances. For instance, the fruit bat *E.* *helvum* can migrate up to 3000 km [93], while some individuals of frigatebird (*F.* *magnificens*) can move over 4400 km from their breeding sites [94]. Thus, they can be considered as “flying bridges” [91] to disperse antimicrobial resistant bacteria even between continents, as shown for Franklin’s gull [95].

**Table 2:** Contamination of bushmeat or wildlife with antimicrobial resistant bacteria

Bacterial species	Wildlife	Resistance genes	Year	Country	Samples (*n*)	Prevalence [% (*n*/*N*)]	References
ESBL*-Escherichia* *coli*	Bats	*bla*_CTX-M-15,_ *bla*_SHV-11_	2017	Gabon	Faeces (68)	9% (6/68)	[135]
	Bats	*bla*_CTX-M-15_, *bla*_CTX-M-14_, *bla*_CTX-M-3_, *bla*_CTX-M-55_	2015–2018	Peru	Rectal swabs (388)	5.2% (20/388)	[89]
	Bats	*bla*_CTX-M-15_	Before 2021	Nigeria	Viscera (180)	1% (2/180)	[136]
	Bears	*bla*_CTX-M_	2015–2016	India	Faeces (21)	76% (16/21)	[137]
	Chimpanzees	*bla*_CTX-M-15_	2018	Uganda	Faeces (86)	11% (9/86)	[138]
	Chimpanzees	Not applicable	2012	Côte d’Ivoire	Faeces (43)	0% (0/43)	[139]
	Condor	*bla*_CTX-M-14_, *bla*_CTX-M-55_	2019	Chile	Faecal swabs (27)	63% (17/27)	[140]
	Gorillas	Not applicable	2011	Central African Republic	Faeces (65)	0% (0/65)	[141]
	Gull	*bla*_CTX-M-14_, *bla*_CTX-M-15_	2010	Bangladesh	Faeces (150)	19.3% (29/150)	[142]
	Macaques	Not applicable	2014–2015	Algeria	Faeces (126)	0% (0/126)	[143]
	Owl	*bla*_CTX-M-8_	2018	Chile	Cloacal swabs (5)	60% (3/5)	[144]
	Rats	Not applicable	2018–2019	Iran	Faeces (100)	0%^a^ (0/100)	[145]
	Rats, shrews	Not done	2013	Vietnam	Faeces (234)	0.4% (1/234)	[86]
	Seabirds	*bla*_CTX-M-8_, *bla*_CTX-M-55_	Before 2022	Brazil	Cloacal swabs (204)	2.5% (5/204)	[91]
	Seabirds	*bla*_CTX-M-15_, *bla*_CTX-M-2_, *bla*_CTX-M-22_	2011	Chile	Faecal swabs (124)	54% (67/124)	[95]
	Wildlife	Not done	2018–2019	Sri Lanka	Faeces (47)	4% (2/47)	[146]
	Wild birds	*bla*_CTX-M_	2010–2013	Brazil	Faeces (112)	12.5% (14/112)	[92]
	Wild birds	*bla*_CTX-M-15_, *bla*_CTX-M-32_	2012	Nicaragua	Faeces (100)	10% (10/100)	[97]
	Wild birds	*bla*_CTX-M-9_	2010	Mongolia	Cloacal swabs (91)	6% (5/91)	[147]
	Wild birds	*bla*_CTX-M-14_, *bla*_CTX-M-15_, *bla*_CTX-M-24_	2015	Mongolia	Cloacal swabs (63)	14% (9/63)	[96]
	Wild boars	*bla*_CTX-M-15_	2014–2015	Algeria	Faeces (90)	33% (30/90)	[143]
Methicillin-resistant *S.* *aureus*	Chimpanzees	Not applicable	2008	Côte d’Ivoire	Fruit wadges (21)	0% (0/21)	[148]
	Lemurs	Not applicable	2012	Madagascar	mucous membrane (25)	0% (0/25)	[148]
	Macaques	*mecA*	2017	Nepal	Saliva (59)	7% (4/59)	[99]
	Marsupials	Not applicable	2018–2019	Brazil	Faeces (23)	0% (0/23)	[149]
	Monkeys	Not applicable	Before 2011	Gabon	Nose (16)	0% (0/16)	[150]
	Rodents	Not applicable	2018–2019	Brazil	Faeces (136)	0% (0/136)	[149]
	Seabirds	*mecA*	Before 2021	Brazil	Cloacal and tracheal swabs (18)	6% (1/18)	[105]

^a^Deduced from third generation cephalosporin resistance

Some migratory birds in the Mongolian desert were colonized with ESBL-producing *E.* *coli* that did not carry the ESBL determinants (*bla*_CTX-M-14_, *bla*_CTX-M-15_, *bla*_CTX-M-24_) on plasmids but on chromosomes, suggesting a more stable integration in the bacterial genome [96].

In addition, there is evidence that bats and pigs are colonized with near-identical ESBL-producing *E.* *coli* [89]. Similarly, ESBL-producing *E.* *coli* from poultry, humans and wild birds in Nicaragua shared the same resistance genes (*bla*_CTX-M-15_), and were detected in same clusters possibly indicating transmission [97].

Despite numerous studies ascertaining colonization, none (in Africa) or very few MRSA (in Latin America and Asia) were detected in free-living wildlife or bushmeat so far (Table 2) [98, 99]. Few studies suggest that *S.* *aureus* can be transmitted from humans to gorillas in captivity, including one fatal case, macaques in temple areas, human-habituated monkeys [100] or chimpanzees living in sanctuaries [99, 101, 102]. This highlights, that wildlife is not only a risk for humans but humans can also be a threat for wildlife particularly if animals are reintroduced into the wild [103]. Noteworthy, MRSA in wildlife can also emerge independently of close contact to humans or anthropogenic antimicrobial selective pressure [104]. For instance, coagulase-negative *Staphylococcus* *sciuri* were identified as the probable reservoir and source of *mecA* in a MRSA isolate from non-migratory seabirds on a remote Brazilian island [105].

### ‘Filth flies’ as reservoirs and vectors for AMR

So called ‘filth flies’ (e.g., *Muscidae*, *Calliphoridae*) belong to the order Diptera (true flies) and are coprophagous insects. By consuming faeces from larger animals and humans, and in this way also bacteria, ‘filth flies’ can be both a reservoir and a vector of antimicrobial resistant bacteria. Thus, ‘filth flies’ could be a link between humans, livestock, wildlife, and bushmeat, particularly in LMIC where adequate sanitation systems are often lacking. The capacity to be a reservoir is, however, limited as the alimentary tract of the flies is a hostile environment to the majority of bacterial species (e.g. *E.* *coli*, *Pseudomonas* *aeruginosa*) [106]. Although the concentration of bacteria decline exponentially during the intestinal passage, defaecation can still be a way of bacterial transmission as bacteria proliferate in faecal droplets [107]. The same is true for regurgitation. The third way of transmission is translocation from the exoskeleton (e.g., insect legs, antennae, labium): approximately 10^3^ of viable bacterial cells (colony forming units) can be transmitted from the exoskeleton per landing [106]. Among antimicrobial resistant species, ESBL-producing *E.* *coli* is the most relevant in ‘filth flies’ in LMIC (e.g. Ethiopia, Nigeria, Thailand, India, Zambia,) with colonization rates between 0.8 and 72.5% (Table 3). Similar to data from humans, CTX-M is the most common ESBL in *E.* *coli* from flies (24.4–100%) [107–110]. It appears that colonization rates with ESBL-producing *E.* *coli* are much higher in the hospital environment than in rural areas as shown in Nigeria and Ethiopia [107, 111]. This suggests that flies could serve as a vector to spread antimicrobial resistant bacteria from the hospital into the community setting. Noteworthy, flies are not only mechanical vectors but could be considered as “reactors of AMR” as resistance genes (e.g. *bla*_CTX-M_, *bla*_CMY-2_) can be horizontally transferred between different bacterial strains within flies [112]. Not only single genes but also combinations of resistance genes can be co-transferred between bacterial cells via plasmids containing *mcr-1* and *bla*_TEM-1_ [109].

**Table 3:** Colonization of ‘filth flies’ with antimicrobial resistant *Escherichia* *coli* and *Staphylococcus* *aureus* in LMIC. *LMIC* Low- and middle-income countries

Bacterial species	Country	Year	Colonization rate in flies, % (*n* of isolates/*n* of flies)	Setting	Fly species (*n*)	References
ESBL-*E.* *coli*	China	2011	3% (37/1228)^a^	Airport	*Chrysomya* *megacephala* (276), *Aldrichina* *graham* (247) and others (705)	[151]
	Ethiopia	2019	6% (5/85)	Hospital, butchery	Not specified (85)	[111]
	Nigeria	2017	0.8% (16/2000)	Urban, semi-urban and rural	Not specified (2000)	[107]
	Thailand	Before 2021	55.7% (334/600)	Urban and rural	*Chrysomya* *megacephala* (600)	[110]
	Thailand	2013–2015	22.6% (53/235)	Urban and rural	houseflies (177), blowflies (32), flesh flies (8), not identified (18)	[109]
	India	Before 2022	11% (17/150)	Milk and meat shops	*Musca* *domestica* (150)	[108]
	Thailand	2018	100% (25/25)	Markets	Not specified (25)	[152]
	Zambia	2015	13.4% (56/418)	Food market	House flies (418)	[153]
Methicillin-resistant *Staphylococcus* *aureus*	Bangladesh	2017–2018	25.3% (101/400)	Hospital	House flies (400)	[154]
	Botwana	2018–2019	1% (10/970)	Hospital	House flies (970)	[155]
	Libya	Before 2015	1.3% (2/150)	Urban	*Musca* *domestica* (150)	[156]
	Nigeria	2017	0.2% (4/2000)	Urban, semi-urban and rural	Not specified (2000)	[106]

MRSA colonization in flies is poorly investigated and varies markedly between geographic regions (0.2–25.3%) in LMIC (Table 3).

### AMR surveillance and integration across sectors

To address the issue of AMR, in 2015, the World Health Organization (WHO) put forward the Global Action Plan for AMR (GAP-AMR) which sets five strategic objectives that aim to reduce the burden of AMR and preserve our ability to treat infections effectively [113]. In these efforts, WHO was joined by the WOAH and the FAO to support countries in strengthening their efforts against AMR. The Tripartite organisations (FAO, WOAH, and WHO) are in the process of developing several tools for monitoring and surveillance. The joint Tripartite AMR country self-assessment survey (TrACSS) tracks country progress towards development and implementation of National Action Plans for AMR [114]. To date, almost 150 countries have joined this initiative and developed National Action Plans for AMR [115]. In 2022, FAO has started developing a global system to aid countries in collecting, analysing and comparing AMR data from animals and food. The International FAO AMR Monitoring (InFARM) platform aims to enable comparisons between settings and facilitate public sharing and harmonisation of AMR data [116]. The FAO has developed an Assessment Tool for Laboratories and AMR Surveillance Systems (FAO-ATLASS) to support countries in strengthening laboratories and improving national AMR surveillance systems for the food and agriculture sectors [117].

The Tripartite Integrated Surveillance System on AMR/AMU (TISSA) is a platform that integrates surveillance data across different organisations and areas. Specifically TISSA is meant to integrate four monitoring and surveillance systems across the three organizations (Global Antimicrobial Resistance and Use Surveillance System (GLASS), WOAH surveillance data on antimicrobial use in animals, ATLASS, and TrACSS) thus encompassing human and animal health, plants, and the environment [118].

To further emphasize the interconnectivity across sectors in the efforts to combat AMR, the WHO is planning the Tricycle project which will integrate surveillance in humans, animals and the environment (Fig. 1). The project is aimed at low-resource countries and focuses on a single pathogen namely ESBL-producing *E.* *coli*. By employing standard methodologies for surveillance and the project will enable participating countries to develop further surveillance systems involving other pathogens and resistance mechanisms [119].

In addition, the Tripartite organizations have developed a One Health priority research agenda to promote scientific interest, increase investment and inform policies related to AMR [120]. One Health provides an integrated and unified approach across sectors aiming to sustainably improve the health of humans, animals and ecosystems [121]. In November 2022, the Antimicrobial Resistance Multi-Stakeholder Partnership Programme was launched in November 2022 by FAO, WHO, WOAH, and the UN Environment Programme (Quadripartite) to strengthen the global efforts regarding One Health integrated surveillance on the human, animal, food and environmental sectors [122].

### Mitigating AMR emergence and transmission

Several measures can be taken to mitigate the emergence and transmission of AMR. Strengthening AMR surveillance and research within but also across sectors can lead to a better understanding of the burden of resistance and enable the implementation of setting-specific measures to prevent further spread. This can only be achieved by increasing diagnostic capacity, by developing reliable integrated systems for reporting AMR data across countries, laboratories and sectors (e.g., including data from human, animal, food, and environmental samples), and by ensuring access to funding for undertaking these activities particularly in LMIC. Of critical importance is also ensuring that data are representative and thus correctly reflect the local/regional AMR landscape. Similarly, reliable data on AMU in animals are needed to inform policy and plan educational interventions for effecting behaviour change and reducing AMU.

Developing new antimicrobial classes and systems (e.g. plasmids, phages), that are used only in food production, would make the emergence of resistance to these antimicrobials less problematic to human health [123]. Further, improving hygiene in animal-rearing facilities, education and veterinary care, as well as adequate waste disposal and treatment of sewage would prevent transmission of AMR determinants between animals, environmental contamination, and entry into the food chain [11]. In addition, research to identify how and where contamination with resistant organisms occurs during food production and commercialization and how it is transmitted to and from humans would aid in developing mitigating interventions.

Vaccines are a promising tool for combating AMR. These act directly by reducing the incidence of infections overall, and thus of resistant infections, and indirectly by reducing AMU. For instance, vaccination of pigs against *Lawsonia* *intracellularis* led to an important reduction in AMU and improved productivity [124] Vaccines have been developed and are being used in aquaculture for preventing infections in high-value Atlantic salmon, however, vaccines for low-value fish which are farmed primarily in LMIC are still needed or underused [125].

Policies and regulations controlling and restricting the use of antimicrobials critical for human health, the uncontrolled purchase of antimicrobials and their use for growth-promotion in animals would lead to safeguarding the future of antimicrobials [11]. Promoting the labelling of animal products according to the use of antimicrobials in food production may also encourage farmers to reduce AMU.

## Conclusions

This review set out to provide an overview on the zoonotic sources of AMR, associated challenges and relevance to LMIC. Livestock farming accounts for substantial AMU in both high- and low-income settings. While regulations to control and restrict excessive use of antimicrobials have been implemented in high-resource settings, efforts should be made to support LMIC in developing strategies to better monitor and optimise AMU. Furthermore, bushmeat and aquaculture are relevant sources of animal meat in LMIC and should be targeted for AMR surveillance and research. Therefore, measures tailored to the specific features of LMIC need to implemented to contain the spread of AMR. Addressing AMR across sectors and settings, will prevent the development and transmission of resistance as well as preserve our ability to effectively treat infections in humans and animals.

## Data Availability

Not applicable.

## Abbreviations

AMR: Antimicrobial resistance
AMU: Antimicrobial use
ATLASS: Assessment Tool for Laboratories and AMR Surveillance Systems
CC: Clonal complex
ESBL: Extended-spectrum beta-lactamases
FAO: Food and Agriculture Organization
GLASS: Global Antimicrobial Resistance and Use Surveillance System
InFARM: International FAO AMR Monitoring
LMIC: Low- and middle-income countries
MRSA: Methicillin-resistant *Staphylococcus* *aureus*
PVL: Panton-Valentine leukocidin
SDG: Sustainable development goals
ST: Sequence type
TISSA: Tripartite Integrated Surveillance System
WHO: World Health Organization
WOAH: World Organization for Animal Health
